# Unravelling the complexities: a scoping review of the collateral effects on bereaved relatives during and beyond the COVID-19 pandemic

**DOI:** 10.1186/s12904-025-01860-w

**Published:** 2025-10-07

**Authors:** M. Nicolai, A. Ullrich, J. Ruck, B. Jaspers, A. Bialobrzeski, R. Degutsch, K. Oechsle, L. Radbruch, I. Gágyor, N. Hettich-Damm

**Affiliations:** 1 Department for Psychosomatic Medicine and Psychotherapy, University Medical Center of the Johannes Gutenberg-University Mainz, Mainz, Germany; 2 Palliative Care Unit, Department of Oncology, Hematology and BMT, University Medical Center Hamburg-Eppendorf, Hamburg, Germany; 3 Department of General Practice, University Hospital Würzburg, Würzburg, Germany; 4 Department of Palliative Medicine, University Hospital Bonn, Bonn, Germany; 5 Department of Palliative Medicine, University Medicine Goettingen, Goettingen, Germany

**Keywords:** Scoping review, Covid-19, Pandemic, Bereaved relatives

## Abstract

The dying phase and the loss of a loved one, as well as the grief that follows, are a difficult process in the lives of relatives. These processes have been exacerbated by the COVID-19 pandemic, as numerous restrictions on contact and care for the dying and deceased have placed an additional burden on relatives. A review was conducted to identify these specific stress factors and their risk factors, as well as support options for bereaved individuals who lost a loved one during the COVID-19 pandemic. The scoping review followed the Joanna Briggs Institute (JBI) methodology for scoping reviews, and the search was conducted in April 2024 (PubMed, Cochrane COVID-19 Study Register, and EBSCO Host, including APA PsychArticles, APA PsychInfo, CINAHL, and Medline). Studies involving adults who had lost a loved one during the official period of the COVID-19 pandemic were included, as well as various quantitative and qualitative study types. Studies that focused exclusively on palliative care and the evaluation of interventions were excluded. Studies were selected according to the Preferred Reporting Items for Systematic reviews and Meta-Analyses (PRISMA) phases. A total of 58 primary studies and five review articles with a total of 118,062 participants met the inclusion criteria and were included in the review. The main findings were that the pandemic and the associated measures placed additional burdens on bereaved individuals and exacerbated mental health problems. Visiting restrictions during the dying phase and restrictions on funerals were perceived as particularly stressful. Participants primarily experienced isolation and loneliness, as well as a lack of professional (e.g., from staff accompanying the dying process and the initial grieving process) and social support (e.g., from family and friends). In addition to personal resources and finding meaning, professional and social support were described as the most important factors in coping with grief during and after the pandemic. Consequently, professional, flexible, and comprehensive support from medical and nursing staff in cooperation with counselling centres and psychologists, as well as promotion of social support through networking services, are key issues for future crises.

## Introduction

The COVID-19 pandemic has led to high numbers of deaths, both due to SARS-CoV-2 infections and other aspects of the pandemic, such as overburdened healthcare systems and delayed diagnoses or treatments [1]. These delays can be seen in connection with psychological mechanisms such as fear of infection on the one hand, and political measures to control infection and prevent the spread of the virus on the other [2]. Both aspects have had a significant impact on people’s daily lives and mental health [3, 4]. These effects have also affected people who were going through particularly difficult phases in their lives. These include relatives who have accompanied a loved one through the dying process and people in mourning [5]. While the end of life, death, and the loss of a loved one are difficult processes even in normal times, they took on a new dimension in the context of the COVID-19 pandemic, as usual farewell rituals, funerals, and memorial services were restricted. These measures, which included strict visiting regulations, restrictions on international travel, and the interruption or restriction of professional services, may have contributed to or exacerbated problems such as social isolation, feelings of loneliness, financial difficulties, and a deterioration in the physical and mental well-being of the bereaved [6].

Previous studies on the grieving process during the pandemic showed changes in grief and emotional responses, as well as negative effects on the health of those affected [7, 8]. In addition, the development of complicated grief disorder and prolonged complex grief disorder (DSM-5-TR and ICD-11) has been observed in connection with restrictions on mourning rituals [9, 10]. The results regarding the emotional benefits or necessity of attending funerals were mixed. However, it was found that the ability of the bereaved to influence the funeral service and create a meaningful farewell were associated with the benefits or necessity of attending funerals [7, 11]. Overall, previous studies have focused on stressful or supportive factors, but have rarely considered both aspects together. Therefore, this review aims to identify additional stressors and sources of support for bereaved individuals who lost a loved one during the official COVID-19 pandemic from March 2020 to May 2023 (WHO). Bereaved individuals are defined as persons who were in a close relationship with the deceased. The study focuses on situations and emotions experienced by relatives before the death of the family member and during the grieving process. This includes aspects of mental and physical health as well as financial, occupational, and social stressors and support factors.

## Methods

The scoping review was conducted as part of the CollPan (Collateral Effects in Pandemics) research project (https://www.netzwerk-universitaetsmedizin.de/projekte/alle-num-projekte), which was carried out within the framework of the University Medicine Network (NUM) and funded by the German Federal Ministry of Education and Research. It was conducted in accordance with the JBI methodology for scoping reviews [12] with the aim of bringing together the literature on the impact of the COVID-19 pandemic on bereaved people worldwide and identifying stressors and support factors. The protocol for the scoping review was also developed in accordance with the JBI methodology for scoping reviews [12] and is available on the OSF research platform (10.17605/OSF.IO/KDHXZ).

### Inclusion and exclusion criteria

Studies were included if they examined the impact of the COVID-19 pandemic on bereaved individuals who had lost a loved one during the official period of the COVID-19 pandemic (March 2020 to May 2023). All studies whose sample included these individuals were included. However, the samples did not have to consist exclusively of related bereaved individuals. For example, if part of the sample consisted of friends who had lost a loved one, that study was also included. Only studies involving adults were included in the analysis. The following types of publications were included in the scoping review: quantitative and qualitative cross-sectional and longitudinal studies, case studies, randomized/quasi-randomized controlled trials, cohort studies, surveys, reviews, and meta-analyses. Only studies in English and German were considered. Studies were excluded if the participating relatives were younger than 18 years of age or if the deceased persons did not die during the pandemic. In addition, studies that focused exclusively on palliative care without including the phase of death and grief after death were excluded. Studies that evaluated interventions for bereaved relatives were also excluded. Reports and position papers were not considered.

### Search procedure

The search was conducted on April 12, 2024. To obtain only records related to COVID-19, the search was limited to articles published between 2020 and 2024. The following databases were searched: PubMed, Cochrane COVID-19 Study Register, and EBSCO Host, including APA PsychArticles, APA PsychInfo, CINAHL, and Medline. A two-step methodology was used. First, an initial limited search was conducted using a title and abstract search to identify articles on the topic and additional search terms. This was followed by a comprehensive search using the original search terms and the additional terms identified in step 1 of the search methodology. The following search strategy with three focal points (participants: bereaved relatives, concept: bereavement, context: COVID-19 pandemic) was used: Relatives OR family OR “significant other” OR spouse OR partner AND mourn* OR grief OR griev* OR bereave* AND COVID-19 OR SARS-CoV-2 OR corona* OR pandemic. To limit the search to relevant articles, a filter was applied in the respective databases for studies involving humans, the publication date (2020–2024), the type of source, and age (adults). The search strategy was applied to all included databases.

### Study selection

**Fig. 1:**
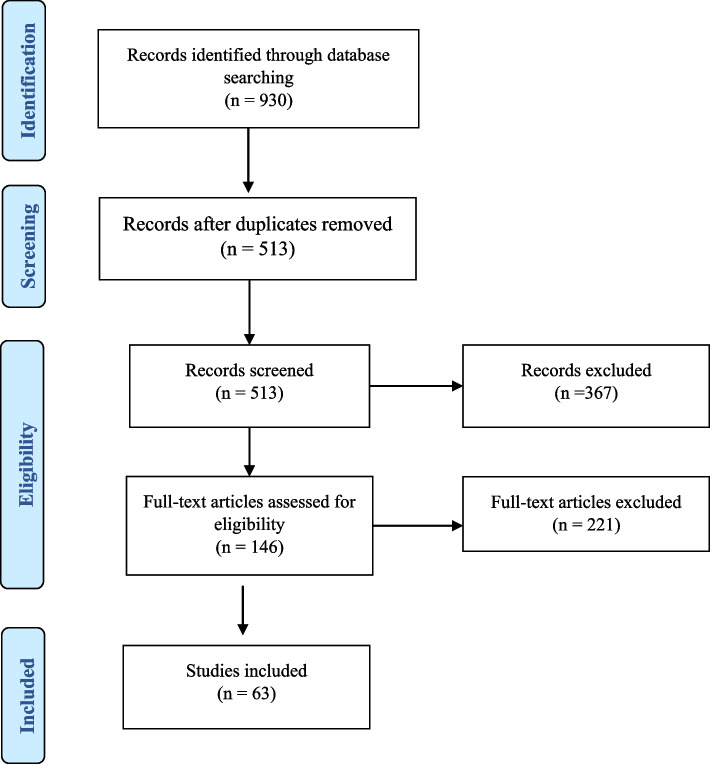
Flow chart of the literature search and study selection

The flowchart (Fig. 1) shows the search and selection process. The initial search yielded 930 records through database searches (EBSCOhost = 333, PubMed = 313, Cochrane = 284). Duplicate records were automatically removed using Rayyan [13] and manually by a reviewer (*n* = 417). After removing duplicates, the titles and abstracts were screened (*n* = 513), with two authors independently reviewing 50% of the records. A total of 367 articles were excluded due to non-compliance with the inclusion criteria, and 10% of all records were reviewed twice in blind mode to ensure author agreement. In three studies, the authors arrived at different assessments (agreement κ≈ 0.869). The disagreements were discussed in consensus meetings. After reviewing the titles and abstracts, 146 records remained for full-text review, with two authors reviewing 50% of the records each. Approximately 10% of the *n* = 146 articles were double-blind reviewed by both authors to ensure author agreement. In one study, the author group arrived at different assessments (agreement κ≈1). The disagreement was resolved in a consensus meeting. A total of 58 articles and five review articles met the inclusion criteria.

### Extraction procedure

The extraction was carried out in parallel by two authors using a pre-developed, tested, and standardized data sheet. The information to be extracted was: author (year), country, study design, time of data analysis, characteristics of the sample (size, age, gender, degree of kinship to the deceased, place of death, cause of death, time since loss), main topic, result (stressors, support factors, special population group or location). The two reviewers exchanged information on the extraction process on an ongoing basis.

## Results

A total of 58 primary studies and five review articles met the inclusion criteria and were included in the scoping review.

### Study characteristics

Most studies (*n* = 24) were conducted in European countries [8, 11, 14–35]. Thirteen studies were conducted in North America [36–48], nine in Iran [5, 49–56], two in Australia [57, 58], three in New Zealand [59–61], four in India [62–65], two in China [66, 67], three in South America [7, 68, 69], one in Indonesia [70], one in Singapore [71], and one in Saudi Arabia [72]. A qualitative cross-sectional analysis of interviews was conducted in 26 studies [15, 16, 18, 19, 25, 30, 32, 33, 37, 39, 44, 49, 50, 52–54, 56, 59–62, 64, 65, 68, 70, 72], one study used a qualitative longitudinal interview approach [31], four studies analyzed documents (e.g., audio diaries, Twitter reports, hospital records) using a qualitative approach [27, 34, 45, 69], and one study was a qualitative case study [42]. Quantitative cross-sectional methods were used in 21 studies [17, 20–23, 26, 28, 29, 34, 36, 38, 40, 41, 43, 47, 51, 55, 57, 58, 66, 67] and two studies used quantitative longitudinal data [14, 46]. Two studies used a mixed-methods approach [24, 63], one study was a randomized controlled trial [48], and five studies were review articles [5, 7, 8, 35, 71]. Most of the data were collected in the early stages of the pandemic. Nineteen primary studies collected data in the first half of 2020 [20, 20, 24, 26, 27, 31, 37–39, 41, 44, 45, 48, 52, 53, 55, 57, 59, 69] and 20 studies in the second half of 2020 [14, 18, 20, 22–26, 28, 29, 34, 39, 41–44, 54, 66–68]. Data collection for 12 studies took place in the first half of 2021 [19, 29, 33, 40, 43, 44, 49, 50, 58, 63, 68, 70] and for three studies in the second half of 2021 [31, 58, 60]. In 2022, data were collected for six studies [47, 48, 51, 57, 58, 61]. Overall, data were collected for 13 studies at several of the above-mentioned time points [20, 24, 26, 29, 39, 41, 43, 44, 48, 57, 58, 68], and 13 studies did not specify the exact time of data collection [15–17, 21, 30, 32, 36, 46, 56, 62, 64, 65, 72]. Regarding the duration of data collection, the average collection period, as determined from 43 studies, was four months.

### Sample characteristics

A total of 118,062 individuals were included in the *n* = 58 primary studies. The smallest sample size was one participant [42], while the largest sample size was 55,796 participants [26]. A total of 30 studies reported the average age of their participants, which was 45.95 years. In *n* = 50 studies, mixed samples were analyzed with regard to gender. Two studies examined women exclusively [42, 72]. Only seven studies reported gender categories other than female and male [20, 22, 34, 36, 40, 47, 58]. Ten studies did not provide any information on gender [5, 7, 8, 23, 28, 33, 43, 51, 69, 71]. A total of 36,994 women, 27,888 men, and 29 people who identified as diverse were examined.

In terms of the relationship to the deceased, 43 studies included spouses or partners, 34 studies included parents, 32 studies included children, 28 studies included siblings, 26 studies included other relatives, 16 studies included grandchildren, 16 studies included friends of the deceased, and five studies did not specify the relationship to the deceased. A total of 57,370 spouses or partners, 2,456 children, 1,187 parents, 538 grandchildren, 413 siblings, 787 other relatives, 564 friends, and 647 individuals with other relationships to the deceased were examined. Only 20 studies specified the average time since the loss, which was 6.55 months. A further 13 studies specified time periods since death ranging from 48 h to 24 months. Three studies specified unspecific time periods and 22 studies did not provide any information at all about the time of the loss. The deceased died mainly in hospitals (*n* = 3,803), at home (*n* = 1,131), in nursing homes (*n* = 872), or in a hospice (*n* = 111). However, 29 studies did not provide any information on the place of death. In most studies (*n* = 19), COVID-19 or other causes were cited as the cause of death. However, 16 studies only included relatives of deceased individuals who had died from COVID-19 or related complications, while one study only included relatives of deceased individuals who had died from causes other than COVID-19. In addition, 22 studies did not specify causes of death. A total of 5,090 people died from COVID-19 or related complications and 56,066 died from other causes. Table 1 provides an overview of the studies and the characteristics of the samples, sorted by study type and alphabetically.

**Table 1:** Overview of the included studies and the characteristics of the samples

First author, year	Country	Study design	Study type	Time of data collection	Sample size/studies	Sample age	Sample gender	Degree of relationship to deceased person	Time sience loss (months)	Location of death	Causes of death
Qualitative cross-sectional interview studies											
Abdekhodaie, 2023 [49]	Iran	Qualiative	Descriptive phenomenological method with in-depth interviews	Feb. to Apr. 2021	30	16–69	f: 19; m: 11	Spouses 8; Parents 9; Siblings 6; Childen 7	> 6 months	na	na
Arnout, 2023 [72]	Saudi Arabien	Qualiative	Qualitative case study design with in-depth interviews	na	10	47–54	f: 10 (100%)	Husband 4; parent 6	na	na	na
Asgari, 2023 [50]	Iran	Qualiative	Colaizzi analysis method with semi-structured interviews	Jan. to Feb. 2021	15	M = 35.13; SD = 13.50	f: 8; m: 7	Spouse 4; parent 6; sibling 1; child 4	na	na	100% COVID-19
Becqué, 2022 [15]	Netherlands	Qualiative	In-depth content analysis with in-depth interviews	na	25	20–79	f: 20 (80%); m: 5 (20%)	Spouse/partner 5 (20%) Adult child 16 (64%) Sister 1 (4%) Adult grandchild 1 (4%) Daughter-in-law 1 (4%) Cousin 1 (4%)	During pandemic	Hospital 11 (42%); Mental hospital 1 (4%); Nursing home 11 (42%); Hospice 1 (4%); Sheltered house 1 (4%); Home 1 (4%)	COVID − 19 Yes 16 (62%) Probably 4 (15%) No 6 (23%)
Bradford, 2022 [60]	New Zealand	Qualiative	Template analysis with in-depth semi-structured inter views	Jul. to Nov. 2021	26	na	f: 21; m: 5	children	na	na	na
Cipolletta, 2022 [16]	Italy	Qualiative	Thematic analysis with interviews	na	20	M = 40; SD = 13.9	f: 15; m: 5	Adult children 16, grandchildren 3, spouse 1	1 to 3 months	na	na
Dennis, 2022 [37]	Canada	Qualiative	Qualitative descriptive study with semi-structured interviews	Mar. to Jul. 2020	28	M = 55.5; SD = 12.0	f: 22 (78.6%)	Child 12 (42.9%); parent 4 (14.3%); friend 4 (14.3%); sibling 3 (10.7%); partner/spouse 2 (7.1%); relative by marriage (in- law) 2 (7.1%); Grandchild 1 (3.6%)	6 to 16 months	ICU 23 (82.1%); COVID- 19 ward 2 (7.1%); Acute medical stepdown unit 1 (3.6%); Palliative care suite 1 (3.6%); Hospital medical ward 1 (3.6%)	na
Dew, 2022 [59]	New Zealand	Qualiative	Qualitative narrative research with semi-structured interviews	Mar. to May 2020	10	20–79	f: 7 (70%); m: 3 (30%)	Spouse/partner (*n* = 2), parent (*n* = 6), sibling (*n* = 1) and grandparent (*n* = 1)	na	Hospice 2; Hospital 5; Home 1; Aged care facility 2	na
Erbicer, 2023 [19]	Turkey	Qualiative	Phenomenological method with semi-structured interviews	Jan. and Mar. 2021	9	19–47	f: 6; m: 3	Grandmother 1, Relative 1, Teacher 1, Father 2, Uncle 3, grandfather	3 month to 1,5 years	na	na
Guité-Verret, 2021 [39]	Canada	Qualiative	Interpretative phenomenological approach with in-depth interviews	May to Nov. 2020	20	M = 54.2; SD = 14.7	f: 17; m: 3	Mother 7 (35%); Father 6 (30%); Spouse 5 (25%); Grand-parent 2 (10%)	*r* = 12–237 days; M = 95.4 (SD = 74,6)	Hospital 14 (70%); residence for elderly 6 (30%)	na
Hanna, 2021 [18]	UK	Qualiative	Interpretative qualitative study with semi-structured interviews	Jul. and Dec. 2020	19	20–79	f: 12; m: 7	Spouse/partner (*n* = 4), adult child (*n* = 11), son/daughter in-law (*n* = 2), niece (*n* = 1), grandchild (*n* = 1)	2 to 6 months	Hospital (*n* = 10); General ward (*n* = 3); Intensive care unit (*n* = 4); Coronavirus ward (*n* = 3); Care home (*n* = 9)	na
Khan, 2022 [62]	India	Qualiative	Qualitative data analysis approaches with semi-structured interviews	na	21	M = 37.42; *r* = 22–66	f: 10; m: 11	partent 4; cousin 4; neighbor 4; friend 3; aunt/oncle 4; grandparent 2	na	6 home, 14 hospitals, 1 hotel	100% COVID-19
Kuş, 2022 [33]	Turkey	Qualiative	Phenomenological approach with interviews	Jun. and Jul. 2021	17	M = 37.14; SD = 17.6	na	Spouse 1 5.8; Father 6 35.3; Brother 2 11.8; Grandparent 6 35.3; Uncle/Aunt 2 11.8	M = 6.14 ± 4.6	na	100% COVID-19
Mas’amah, 2023 [70]	Indonesia	Qualiative	Exploratory thematic analysis with semi-structured interviews	May 2021 to Jul. 2021	10	18–28	f: 8; m: 2	children	last 12 months	na	100% COVID-19
Mohammadi, 2021 [53]	Iran	Qualiative	Content analysis design with semi-structured interviews	Feb. to May 2020	16	M = 38	f: 9; m: 7	6 wives, 3 husbands, 5 chil- dren, and 2 mothers	na	hospital all	100% COVID-19
Mojarad, 2021 [56]	Iran	Qualiative	Graneheim and Lundman’s qualitative approach with semi-structured interviews	na	16	20–60	m: 6 (37.5%); f: 10 (62.5%)	Mother 1(6.25); Sister 2 (12.5); Brother 1 (6.25); Daughter 8 (50); Son 4 (25)	Within the last week	na	100% COVID-19
Mondal, 2024 [65]	India	Qualiative	Qualitative research approach with in-depth interviews	na	12	M = 41,83	f: 50%	6 children; 3 spouse; 2 Grandchild; 1 parent	> 1 year	hospital	COVID-19 complications
Morgan, 2023 [61]	New Zealand	Qualiative	Reflexive thematic analysis with in-depth interviews	Feb. and Jun. 2022	30	40 s-80 s	f: 22; m: 8	Spouse 11; Child 12; Sibling 6; Neighbour 1	na	na	all no COVID
Mortazavi, 2023 [52]	Iran	Qualiative	Phenomenological study with interviews	Apr. to Jul. 2020	15	M = 39; SD = 12.8	f: 9; m: 6	6 children, 4 spouses, 3 niece/nephew, 1 brother in law, 1 brother	na	na	na
Moya-Salazar, 2022 [68]	Peru	Qualiative	Qualitative approach with interviews	Nov. 2020 and Jun. 2021	15	*r* = 20–72	f: 10; m: 10	parent 8, grandparent 3, spuse 1, uncle/aunt 2, sibling 1	2–16 month	na	100% COVID-19
Ostadhashemi, 2022 [54]	Iran	Qualiative	Descriptive phenomenological approach with in-depth interviews	Sep. and Dec. 2020	18	*r* = 30–78	f: 8; m: 10	spouse 5, parent 4, child 8, sister 1	na	na	100% COVID-19
Patel, 2022 [64]	India	Qualiative	Content analysis with interviews	na	10	M = 40.5; SD = 8.40; *r* = 26–52	f: 2; m: 8	spouse 1, nephew 1, children 8	1 month	hospital all	100% COVID-19
Pauli, 2022 [25]	Germany	Qualiative	Descriptive-interpretive qualitative study with semi-structured interviews	Aug. to Nov. 2020	32	40–89	f: 27; m: 5	Children 25; Spouse/Partner 5; Sibling 1; Son/daughter in-law 1	M = 5	Hospital 10; Nursing home 10; Hospice 5 Home 5; Reha facility 1; Palliative care unit 1	COVID 4; no COVID 28
Testoni, 2021 [30]	Italy	Qualiative	Thematic analysis with interviews	na	40	M = 47; SD = 9.85; *r* = 23–63	f: 80%	22 father, 16 mother; 3 grandparents, 3 spouse, 2 brother	na	na	na
Vachon, 2023 [44]	Canada	Qualiative	Interpretative phenomenological analysis with interviews	May 2020 and Mar. 2021	20	M = 54; *r* = 73–21	f: 19; m: 1	Mother 8; Father 7; Sibling 1; Spouse 3; Grandparent 1	*r* = 12–260 days	Hospital 14; Residence elderly 6	na
Vieveen, 2023 [32]	Netherlands	Qualiative	Thematic analysis with interviews	na	17	55–84	f: 14 (82%); m: 3 (18%)	spouses	2–12	na	na
Qualitative longitudinal interview study											
Nierop-van Baalen, 2023 [31]	Netherlands	Qualiative	Inductive content analysis with interviews	May 2020 and Sep. 2021	10	na	f: 9; m: 1	3 spouses, 7 children	na	hospital 6, home 1, nursing home 3	na
Qualitative cross-sectional analysis of documents											
Menichetti Delor, 2021 [27]	Italy	Qualiative	Thematic analysis with written reports	Mar. to Jun. 2020	246	na	f: 133 (54%); m: 113 (46%)	Child 132 54%; partner 56 23%; Sibling 21 9%; Nephew 12 5%; other	48–72 h	hospital	100% COVID-19
de Oliveira, 2024 [69]	Brasil	Qualiative	Inductive thematic analysis with digital media documents	Mar. to Apr. 2020	23	na	na	Children 6; cousins 4; mothers 3; daughters-in-law 3; nephews 2; husbands 2; wife 1; brother-in-law 1; grandson 1	na	na	na
Selman, 2021 [34]	UK	Qualiative	Qualitative content analysis with tweets	Apr. 2020	191	na	na	Parent 39; Uncle 14; Aunt 13; Brother/in-law 6; Sister/in-law 2; Cousin 4; Grandparent 33; Spouse 2; other	na	Hospital 43; nursing home 15; Home 6; Hospice 1; other	na
Tay, 2021 [45]	USA	Qualiative	Content analysis with audio diaries	Mar. and May 2020	6	M = 56.80; SD = 14.22; *r* = 32–67	f: 5 (83.33%)	Spouses (*n* = 2), adult children (*n* = 3), sibling (*n* = 1)	M = 4.17 (SD = 1.94) *r* = 2–7	hospices	na
Qualitative cross-sectional case study											
Hinkson, 2022 [42]	USA	Qualiative	Qualitative case study method	Dec. 2020	1	na	f	parent	8 months	hospital	na
Quantitative cross-sectional surveys											
von Blanckenburg, 2023 [29]	Germany	Quantitative	Paper-based survey	Nov. 2020 and Jul. 2021	142	M = 58.89; SD = 14.31; *r* = 18–88	m: 84 (59.2%)	Partner 49 (35.5); Sibling 6 (4.3); Son/daughter 63 (45.7); Extended family 20 (14.4)	6	hospital	COVID-19 26 (18.2%)
Breen, 2021 [38]	USA	Quantitative	Online survey	early Nov. 2020	307	M = 35.58; SD = 10.66	f: 49.2%; m: 50.8%	mediate family (25.4%); extended family (22.5%); romantic partner (12.4); acquaintance (20.2%); close friend (18.6%); or other (1.0%)	< 1 to 6 months or more	na	100% COVID-19
Carson, 2021 [17]	UK	Quantitative	Online survey	na	185	16–44: 50; 45–64: 123; > 65: 12	f: 171; m:14	na	na	na	100% COVID-19
Chen, 2022 [36]	USA	Quantitative	Online survey	na	519	M = 49.1; SD = 19.9; *r* = 19–69	f: 179 (34.5%); m: 337 (64.9%); d: 3 (0.6%)	Spouse/partner 28 (5.4%); Parent 76 (14.6%); Grandparent 150 (28.9%); Child 4 (0.8%); Sibling 13 (2.5%); Aunt/uncle 48 (9.2%); Cousin 53 (10.2%); Niece/nephew 7 (1.3%); Friend 127 (24.5%); Other 2 (0.4%); No response 2 (0.4%)	M = 6.4 months (SD = 4.3)	na	COVID-19 (56%)
Chen, 2021 [66]	China	Quantitative	Online survey	Sep. to Oct. 2020	422	M = 32.73; SD = 9.31	f: 188 (44.5%); m: 234 (55.5%)	Partner 139 (32.9%) Child 24 (5.7%) Parent 97 (23.0%) Grandparent 69 (16.4%) Relative 22 (5.2%) Friend 64 (15.2%) Other 7 (1.7%)	na	na	COVID-19 408 (96.7%) COVID-19 related complicationd 14 (3.3%)
Downar, 2022 [41]	canada	Quantitative	In-person survey	Mar. and Aug. 2020	121	M = 58.4; SD = 14.7	f: 80; m: 41	Spouse/partner 24; Child 75; Sibling 9; Friend 2; other 11	na	hospital	30
Drucker, 2023 [21]	Spain	Quantitative	Online survey	na	104	M = 31	f: 82 (78.8%); m: 22 (21.2%)	Most close 25 (24%) Close 39 (37.5%) Else a 40 (38.5%)	M = 10.75 (SD = 5.85)	na	COVID-19 29 (27.9%); Other causes 75 (72.1%)
Gang, 2022 [43]	USA	Quantitative	Online survey	Oct. 2020 and Jul. 2021	1470	na	na	Parent 477 (32.4%); Offspring 203 (13.8%); Sibling 102 (6.90%); Extended Family 186 (12.7%); Friend 111 (7.60%); Other 391 (26.6%)	24.5 (84.4) months	na	COVID-19 118 (8.1%)
Harrop, 2021 [20]	UK	Quantitative	Online survey	Mar. and Dec. 2020	711	M = 49.5; SD = 12.9; *r* = 18–90	m: 74 (10.4%); f: 628 (88.6%); d: 7 (1.0%)	Partner 152 (21.4%); Parent 395 (55.6%); Grandparent 54 (7.6%); Sibling 23 (3.2%); Child 15 (2.1%); Other family (6.5%); Colleague or friend 26 (3.%)	Md = 5 months; *r* = 1–279 days	Hospital 410 (57.7%); Home 158 (22.2%); Hospice 37 (5.2%); In a care home 91 (12.8%) Other/Don’t know 13 (1.8%)	COVID-19 43.8% (*n* = 311); cancer 21.9% (*n* = 156); other 16.7% (*n* = 119)
Katzman, 2022 [40]	USA	Quantitative	Online survey	Feb. to May 2021	146	M = 34.34; SD = 9.31; *r* = 19–65	f: 83 (56.8%); m: 60 (41.1%); d: 1 (0.0%)	Children 65; sibilings 56; spouses 20; parents 5	na	na	100% COVID-19
Khalafi Kasalani, 2023 [51]	Iran	Quantitative	In-person survey	2022	160	20–81 +	na	Spouse 37 (23%); Children 69 (43%); Siblings 27 17%); Parents 27 (17%)	na	na	na
Lobb, 2024 [57]	Australia	Quantitative	Online survey	Jan. 2020 and Feb. 2022	744	M = 56.1; SD = 11.5	f: 610 (93.7%)	Partner 315 (42.3); Parent 175 (23.5); Sibling 80 (10.8); Child 24 (3.2); Other family 94 (12.6); Friend 56 (7.5)	M = 10.0 (SD = 6.0)	hospital 514 (69.1) home 230 (30.9)	COVID-19 related 11 (1.5%)
Maccallum, 2024 [58]	Australia	Quantitative	Online survey	Apr. 2021 to Apr. 2022	1911	M = 55.18; SD = 12.11	f: 94.8%; m: 4.7%; d: 0.3%	Parent 45.1%; Partner 17.2%; Child 7.4%; Sibling 9.3%; Other family member 14.0%; Close friend 7.0%	M = 10.07 (SD = 5.89)	Home 26.3%; palliative 16.9%; ICU 14.3%; hospital ward 18.7%; Care facility 19.4%; Other 4.4%	COVID-19 2.9%
Mayland, 2021 [22]	UK	Quantitative	Online survey	Jun. to Sep. 2020	278	M = 53.4	f: 216 (78.0%); m: 59 (21.3%); d: 2 (0.8%); m = 1 (0.4%)	Children 174 (62.6%); partner 22 (7.9%); Parent 4 (1.4%); Son-in-law/daughter-in-law 12 (4.3%); sibling 6 (2.2%); Niece/nephew 13 (4.7%); Grandchild 19 (6.8%); Friend 14 (5.0%); Neighbour 1 (0.4%); Other 13 (4.7%)	na	Hospital 75 (27.1%); Home 30 (10.8%); Nursing care or residential home 162 (58.5%); Hospice 10 (3.6%)	COVID certainly 82 32.0%; COVID probably 28 11.0%; COVID probably not 54 21.1%; COVID certainly not 92 35.9%
Schneider, 2023 [47]	USA	Quantitative	Online survey	Jan. and Feb. 2022	196	M = 19.29; SD = 2.08	f: 133 (67.9%); m: 61 (31.1%); d: 2 (1%)	Grandparent, n (%) 89 (45.4); Close friend 36 (18.4); Aunt/uncle 33 (16.8); Parent 12 (6.1)	last 2 years	na	na
Selman, 2022 [28]	UK	Quantitative	Online survey	Aug. 2020 to Jan. 2021	711	M = 49.5; SD = 12.9; *r* = 18–90	m: 74 (10.4%); f: 628 (88.6%); d: 7 (1%)	Partner 152 21.4%; Parent 395 55.6%; Grandparent 54 7.6%; Sibling 23 3.2%; Child 15 2.1%, other family 46 6.5%; friend 26 3.7%	Md = 152 days; *r* = 1–279	Hospital 410 57.7%; Home 158 22.2%; Hospice 37 5.2%; care home 91 12.8%; Other 13 1.8%	311 (43.8%) COVID-19
Shahini, 2022 [55]	Iran	Quantitative	Online survey	Jun. to Aug. 2020	75	M = 33.05; SD = 19.3; *r* = 15–78	f: 18 (54.5%) and 11 (26.2%); m: 15 (45.5%) and 31 (73.8%)	first degree relative 9 27.3% and 1 2.4%, second 24 72.7% and 41 97.6%	30 days to 3 months	hospital	COVID 33; non-COVID 42
Tang, 2021 [67]	China	Quantitative	Online survey	Sep. and Oct. 2020	422	M = 32.73	m: 56%	partner (33%; *n* = 139), child (6%; *n* = 24), parent (23%; *n* = 97), grandparent (16%; *n* = 69), other relative (5%; *n* = 22), friend (15%; *n* = 64), other (2%; *n* = 7)	M = 5.10; SD = 1.72	na	COVID-19 (97%; *n* = 408); COVID-19-related complications (3%; *n* = 14)
Wang, 2022 [23]	Europe data from 27 countries	Quantitative	Phone survey	Oct. 2019 to Mar. 2020 and Jun. to Aug. 2020	55,796	50–80 +	f: 54%	spouses	na	na	na
Wang, 2022 [26]	Europe	Quantitative	Phone survey	Jun. to Aug. 2020	51,383	*r* = 50–104	na	na	na	na	1,363 COVID-19
Quantitative longitudinal surveys											
Becqué, 2023 [14]	Netherlands	Quantitative	Online survey	Nov. 2020 to Jan. 2021	200	M = 57; SD = 11; *r* = 26–88	f: 169 (84.5%); m: 31 (15.5%)	Child 136 (68.0%) Partner 34 (17.0%) Other relative 30 (15.0%)	First wave of pandemic	Nursing home 84 (47.2%); Hospital 51 (28.7%); Home 23 (12.9%); Hospice 12 (6.7%); Other 8 (4.5%)	Confirmed/suspected COVID-19 103 (57.9%) No confirmed/suspected COVID-19 75 (42.1%)
Lapenskie, 2024 [46]	canada	Quantitative	Phone survey	na	111	M = 58.2	f: 67.7%	Spouse/partner 21 18.9 Child 69 62.2 Sibling 8 7.2 Friend 2 1.8 Other 11 9.9	we contacted them 12 to 18 months after their family member’s death	hospital	30 COVID + ve, 46 COVID − ve, and 45 pre-COVID
Quantitative and qualitative cross-sectional studies											
Majid, 2022 [63]	India	Quantitative & qualitative	In-person survey & semi-structured interviews	Mar. to Feb. 2021	89	na	f: 34; m: 55	family member	na	na	37 COVID-19; 52 other
Renckens, 2024 [24]	Netherlands	Quantitative & qualitative	Paper-based survey & thematic analysis with interviews	Dec. 2019 to Feb. 2020; Mar. to May 2020; Oct. 2020 to Jan. 2021	90	30–66 +	f: 63 (70.0%); m: 27 (30.0%)	Partner 45 (50.0%); Children 33 (36.7%); Other 12 (13.3%)	na	ICU	na
Randomized controlled trial											
Stahl, 2024 [48]	USA	Quantitative	Randomized controlled trial	Jan. 2020 and Dec. 2022	80	M = 70.4; SD = 6.6	f: 80%	spouses or life partners	M = 6.1 (SD = 3.4)	na	COVID-19 out of 80
Reviews											
Braz Sola, 2023 [7]	Brasil		Meta-synthesis of qualitative studies	March 2020 to December 2021	14	20 to 83	na	88 children, 49 friends, 38 partners, 38 grandchildren, 34 nephew/nieces, 21 parents, 18 siblings, and others	During the pandemic	87 hospitals, 41 nursing homes, 18 ICU, 12 home, 2 hospice, 4 other	COVID-19 being the main cause of death
Firouzkouhi, 2023 [5]	Iran		Integrative review	May to Jan. 2021	15	na	na	na	na	na	na
Hasdenteufel, 2022 [35]	France		Systematic review	February 2022	18	M = 58.13	f: 68.96%	Spouses (55.39%)	na	na	na
van Schaik, 2022 [8]	Netherlands		Overview Review	Jan. 2020 to Dec. 2021	28	na	na	na	na	na	na
Tao, 2022 [71]	Singapur		Scoping review of qualitative studies	Aug-21	69	na	na	na	na	na	na

### Burden and support factors identified

**Fig. 2:**
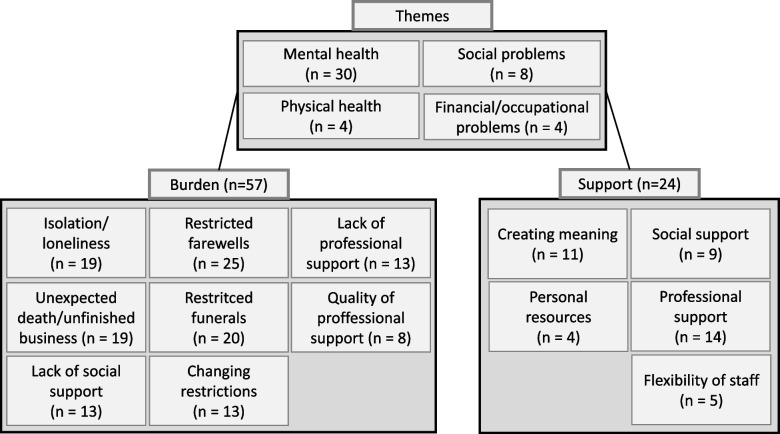
An overview of overarching themes as well as burden and support factors

An overview of overarching themes and stressors and support factors is presented in Fig. 2. A total of 57 primary studies reported stressors, while only 24 studies reported support factors. Overarching themes related to stressors and support were mental health in 30 studies [14, 17, 19–23, 25–27, 29, 30, 34, 36–38, 40, 48–51, 56–58, 60, 62, 66, 67, 70, 72], social problems in eight studies [20, 42, 44, 50, 53, 58, 62, 72], physical health [32, 50, 56, 72], and financial or occupational problems in four studies each [50, 53, 58, 62]. In 25 studies restrictions related to bereavement [15, 16, 18, 19, 25, 27–31, 33, 34, 36, 37, 42, 50, 53, 56, 59, 60, 63–65, 68, 69] and in 20 studies restrictions related to funerals [16, 19, 27, 29, 33, 34, 36, 37, 41, 42, 50, 52, 53, 56, 63–65, 68–70] were mentioned as burdens. In addition, isolation and loneliness [15, 20, 25, 28, 33, 39, 45, 48–50, 57, 61–65, 68, 69, 72] as well as the unexpected death or unfinished business with the deceased were reported in 19 studies [14, 16, 19, 22, 27, 28, 34, 42, 43, 48, 50, 53, 54, 56, 63, 65–67, 69] as important burdens. Similarly, in 13 studies the lack of professional support [16, 20, 25, 28, 29, 32, 42, 45, 50, 57, 59, 63, 64] and in 8 studies its inadequate quality [15, 18, 22, 28, 30, 54, 60, 64] as well as in 13 studies a lack of social support [20, 28, 31, 34, 41, 45, 50, 52, 59, 60, 62, 63, 70] were also perceived as burdensome. The uncertainty surrounding COVID-19 and the changing restrictions to prevent the spread of the virus were also perceived as stressful which was described in 12 studies [15, 22, 25, 29, 32, 50, 54, 58–61, 72]. In 14 studies pprofessional support [15, 18, 19, 22–25, 27, 37, 45, 49, 50, 60, 66] and in 5 studies the flexibility of specialist staff despite the existing restrictions [18, 22, 25, 37, 60] were cited as supportive factors. In addition, the creation of meaningful moments or finding meaning as part of post-traumatic development was identified as a supporting factor in 11 studies [15, 16, 27, 30, 32, 36, 37, 39, 45, 49, 66]. In 9 studies social support [16, 19, 22, 30, 32, 34, 45, 50, 72] and in four studies personal resources such as traditions and rituals, faith and spirituality, or mindfulness [19, 42, 50, 72] were also supportive for bereaved relatives.

Being the spouse or partner of the deceased or having had a close relationship with them was frequently cited as a risk factor for higher levels of stress, more intense grief, or poorer mental health [14, 21, 28, 29, 32, 34, 38, 43, 48, 58, 66, 67, 72]. In addition, some studies found that caring for and dying at home were associated with higher levels of distress [34, 57, 59], while other studies concluded that deaths in hospitals and nursing homes were more distressing [22, 28, 34]. Risk factors included the younger age of the deceased, for example (young) children [58, 66], or the older age of the bereaved, for example widowhood in older couples [26, 48]. The included reviews (*n* = 5) also showed that pandemic restrictions in particular were perceived as stressful. Before death, these restrictions included limited visiting hours, absence at the time of death, and routines that were not possible. After death, these restrictions affected the funeral and rituals of (shared) mourning. Emotional and psychological difficulties were also mentioned, such as fear of contracting COVID-19 or fear of death. Mental health problems were mainly loneliness, helplessness, guilt, and anger. Social problems, especially social isolation and stigmatization, as well as financial burdens, were also identified. In contrast, communication, finances, counseling, education, and spiritual support were mentioned as coping strategies. A more detailed description of the main themes and results per study is presented in Table 2, sorted by study type and alphabetically.

**Table 2:** Overview of main themes and results of included studies

First author (Year)	Main topic	Burden	Support	Most affected persons	Importance of location of death
Qualitative cross-sectional interview studies					
Abdekhodaie, 2023 [49]	experiences of bereavd families	Complicated and prolonged grief: pandemic had deep effects on the person and affects his attitude toward the world and existence	Post-traumatic growth, which can only be achieved by searching, discovering, and creating new meaning—professional support	na	na
Arnout, 2023 [72]	breast cancer patients and palliative workers	Risk factors for grief: guilt and self-blame, contradictory feelings, suppressing the emotional responses, withdrawing from social relations, quarantine procedures, tension and fatigue related to diabetes, side effects of breast cancer treatments	Coping strategies: God and Belief, expressing feelings of grief, accept behavior, talking with trusted people, mental and spiritual mindfulness exercises, going out to nature, meditation, walking	emotional attachment to bereaved person	na
Asgari, 2023 [50]	grief experiences	unexpressed grief, psychosomatic reactions, negative emotions, family, social, occupational problems, sleep problem, disrupted vision of the future, depression, loneliness, confusion, stress about illness, anxiety about future, living in limbo (fear and hope), problems with burial and funeral, guilt and remorse, staying in grief condition, shock and denial, financial stress, losing family enthusiasm, losing supporter, distrust of others, doubt about support and assistance of others	Professional psychological support and support of familiy and friends—empathy and companionship, holding funerals	na	na
Becqué, 2022 [15]	Dignity during end-of life care	‘Dealing with an unknown illness’, ‘Being isolated’, ‘Restricted farewells’ and ‘Lack of attentiveness and communication’	Meaningful end-of-life moments’ and ‘Compassionate professional support’	na	na
Bradford, 2022 [60]	Care, connection, and social distancing—baby loss	‘Distanced and Impersonal care’; ‘Navigating Hospital Rules’; ‘Hindered Access to Social Support’; ‘Exclusion of Non-birthing Parent’: The exclusion of non-birthing parents from the care was a particularly difficult aspect contributing to divergent experiences of the loss and additional distress for both parents	‘Continuity of Relational Care’: Flexibility and compassion by healthcare professionals, and particularly the maintenance of continuity of relational care afforded by the extant model of midwifery continuity of care in the country, offered some mitigation of negative effects	na	na
Cipolletta, 2022 [16]	Uncertainty, shock and anger—experiences of loss	suddenness of the death, lack of farewell by means of a funeral prevented participants from realizing the loss and undertaking a meaning-making process, anger let mourners focuss all their attention on denouncing medical and government institutions	use dramatic experience as a turning point, ability to find a silver lining is pivotal to access meaning reconstruction, socially shared grief to make sense of the loss, relied mainly on informal support virtually	na	na
Dennis, 2022 [37]	Sacrifice and solidarity—family experiences of bereavement	Profound loss and enduring grief, fears about the patient’s experience of isolation and changes to postmortem rituals lead to despair and contributed to long-lasting grief	solidarity among clinical staff and experiencingd a sense of unity with staff, frequent, flexible communication, exceptions for family presence when safe, targeted efforts to connect patients whose isolation is intensified by functional impairment or limited technological access, attitude of acquiescence, some framing their experience as a sacrifice made for the public good	na	na
Dew 2022 [59]	experiences of loss, grief, bereavment	Restrictions during death experience; mourning in isolation; difficulties regarding availability of support; having to adjust to rules that were constantly changing, the loss of family and community support	na	na	especially for those who had been caring for a loved one at home
Erbicer, 2023 [19]	delayed business	grief and mourning responses included cognitive, emotional, and behavioral responses; risk factors included the expectation of harm, unfinished business, and restriction of death-related religious-cultural rituals,	protective factors included relative support, tele-support, positive coping strategies (cognitive, behavioral, and religious-spiritual), and delayed business, psychological support services	na	na
Guité-Verret, 2021 [39]	Expressing grief through metaphors	lived and understood their experience in terms of metaphoric cut-offs, obstructions and shockwaves representing the grief process and the bereaved’s quest for social connection, narrative coherence and recognition	patients and families’ connection and encourage the bereaved to tell the story of the death of their loved one in order to make it more consistent and meaningful	na	na
Hanna, 2021 [18]	end of life and bereavement	relied on connecting virtually with their family, health and social care professionals as instrumental to ensuring connectedness between patients and relatives at end of life, increased communication needs, such as more holistic information about their dying family member’s wellbeing, and psychological support, need for practical and emotional support	opportunities to ‘say goodbye’	na	na
Khan, 2022 [62]	Last Honors and Life Experiences of bereaved families	impact of COVID-19 on burial rituals and customs; shades of grief, bereavement care, community response, and coping with loss, bereaved family members were in danger of marginalization, economic burdens, psychological traumas, and overall reduced quality of life	na	na	na
Kuş, 2022 [33]	grief experiences	“death in isolation”, “changing cultural–religious practices that cannot be performed”, “not being able to say goodbye”—transformed grieving process create unique psychosocial needs	na	na	na
Mas’amah, 2023 [70]	Death, Funeral Rituals, and Stigma	the rushed nature of these funerals led to resistance from families and prevented bereaved families from performing the usual cultural and religious funeral rituals, combined with stigma from their neighbors, led these families to have poor psychological wellbeing	na	na	na
Mohammadi, 2021 [53]	mental health	Emotional shock included (feelings of guilt and rumination, bitter farewell, strange burial and concern about unreligious burial), and fear of the future included (instability in the family, lack of job security and difficult financial conditions, Stigmatization and complications in social interactions)	na	na	na
Mojarad, 2021 [56]	Mourning	psychological, behavioral, and physical reactions, virtual mourning, regretful mourning, and feelings of rejection and fear	na	na	na
Mondal, 2024 [65]	Missing Funerals, Death Rituals, and Complicated Grief	mourn in isolation, have been still struggling to come to terms with their grief, death of their loved ones in solitude, improper funerals in their absence, and missing death rituals only have added more misery to the already toilsome grieving experience of losing a close relative suddenly	na	na	na
Morgan, 2023 [61]	end of life care	A key finding was that dying alone and contracting COVID-19 were seen as equally significant risks by bereaved families. Five key themes: (1) compromised connection; (2) uncertain communication; (3) cultural safety; (4) supported grieving and (5) silver linings	na	na	na
Mortazavi, 2023 [52]	Mourning	inability to hold the usual ceremonies for mourning and receive the social support needed in this period, the relatives of the deceased encounter various conditions that disrupt the grieving process and may lead to the spread of unresolved grief in future	na	na	na
Moya-Salazar, 2022 [68]	end of life and grief	death in isolation, the loss of rituals, funeral practices were altered by health provisions	na	na	na
Ostadhashemi, 2022 [54]	Complicated Grief	crisis in crisis, circumstances of death and its consequences, and lack of preservation of patient dignity were extracted as main categories, neglecting grieving families and related issues can lead to delays and difficulties in the process of recovery and intensification of their psychosocial pressures	na	na	na
Patel, 2022 [64]	Tertiary Care Hospital	difficulty in proper communication during hospitalization, disrupted end-of-life and funeral rituals and accepting harsh realities related to the changes; telephonic mode of communication was not sufficient and created doubts related to death—remorseful and deprived of the traditional rituals, dealing with grief alone	na	na	na
Pauli, 2022 [25]	bereaved relatives’ experiences	The lack of information, of support by others and physical closeness due to the visiting restrictions, as well as not being able to say goodbye, were felt as burdens and led to emotional distress, Even months after a death, relatives suffer from a high burden due to missing opportunities to say goodbye and stay in touch, relatives are dependent on the support of healthcare professionals to ensure closeness with the patient and to gain information about their condition	case-by-case decisions were made and creative ways of staying in touch were experienced positively	na	na
Testoni, 2021 [30]	From Traumatic to Ambiguous Loss and the Role of the Internet	abandonment anger and guilt, dehumanized disappeared, derealization and constant rumination, grief had a complex profile: on the one hand, it was traumatic and characterized by all the risk factors causing mourners to experience prolonged grief, but on the other, some features were similar to ambiguous loss (that occurs without closure and clear understanding) because of the impossibility to be with their relatives in their final moments	social support and the importance of sharing photos on Facebook, the use of social networks proved to be a valuable source of support and photographs were a powerful tool in facilitating the process of mourning by encouraging narration and sharing	na	na
Vachon, 2023 [44]	ethical tensions	struggle with multiple responsibilities (collective, relational, and personal), emotional cost of choices, ethical struggles: (1) Flight or fight: Struggling with collective responsibility; (2) Being torn apart: Assuming relational responsibility and (3) “Choosing” oneself: The cost of personal responsibility	na	na	na
Vieveen, 2023 [32]	Meaning-making—spouses	lacking adequate information, personalized care, and physical or emotional proximity; these challenges complicated their experience of a meaningful death of their partner	many interviewees appreciated the exchange of experiences with others and any last moments together with their partner, Bereaved spouses actively sought valuable moments, during and after bereavement, that contributed to the perceived meaning	spouses and partners	na
Qualitative longitudinal interview study					
Nierop-van Baalen, 2023 [31]	Relatives’ grief at three moments	Losses were threefold: the loss of the loved one; of the (desired) way to say farewell, and of social support. We identified five ways in which the three COVID-19 related loss experiences interacted: overshadowed grief, cumulative grief, triggered grief, derailed grief, and conciliatory grief	na	na	na
Qualitative cross-sectional analysis of documents					
Menichetti Delor, 2021 [27]	Phone follow up—families’ grief reactions and clinical psychologists’ roles	without death rituals, solitary, unexpected, unfair, unsafe, coexisting with other stressors, close to a traumatic grief,	families’ needs ranged from finding alternative rituals to giving meaning and expressing different emotions, psychologists played both a social-institutional and a psychological-human role through the calls	na	na
de Oliveira, 2024 [69]	suppressing funeral rituals	suffering experienced by the sudden death of a significant person, which is amplified by the absence or impediment to performing familial farewell rituals, causing feelings of disbelief and indignation	na	na	na
Selman, 2021 [34]	Sadness, despair and anger—twitter data	Sadness, despair, hopelessness and anger, lack of social support, disrupted rituals, sense of political neglect or mistreatment,	encourage positive public health messages, express condolences to and support others, and pay tribute to the deceased, ambivalence about the use of video-conferencing technology	na	na
Tay, 2021 [45]	Analysis of Audio Diaries—Hospice	“isolation” (*n* = 17), defined as being unable or reluctant to access informal or formal social support networks, or feeling alone; “bereavement processes” (*n* = 147), informed by the dual process model of bereavement (restoration and loss-oriented stressors)	“social connection” (*n* = 23), defined as being able to access or seeking informal or formal social support networks; caregivers were able to connect with others despite physical distancing expectations, expressed loneliness and grief while in isolation, and described moving on in the face of uncertainty	na	na
Qualitative cross-sectional case study					
Hinkson, 2022 [42]	Transnational Caregiving and Grief	challenging family dynamics, limited resources of support, feelings of guilt, and the loss of familiar traditions to memorialize affect how grief is experienced, cognitive awareness of the death, yet often with the absence of a body to view or limited funeral rites, leaves sorrow unfinished, life continues but possibly on hold, until some type of funeral or memorial rite can be enacted	distance and employment may also provide an emotional buffer to manage the prolonged grief process	na	na
Quantitative cross-sectional surveys					
von Blanckenburg, 2023 [29]	Prolonged Grief	Prolonged grief was present in 44.4% of the bereaved, 76.2% of the relatives reported feeling distressed due to visitor restrictions, and the majority of them were unable to bid farewell, pastoral or psychological care was also lacking, inability to bid farewell after death, feeling of threat due to the pandemic, depression, and anxiety were significantly associated with prolonged grief	na	low education, emotional closeness, loss of a spouse were significantly associated with prolonged grief	na
Breen, 2021 [38]	Psychological Risk Factors of Functional Impairment	Separation distress, COVID-19 grief, and posttraumatic stress significantly explained functional impairment; most participants’ scores were in the clinical ranges for generalized anxiety, depression, dysfunctional grief, and functional impairment, logistic regression showed that, after controlling for covariates, the odds of functional impairment significantly increased by 27% for higher scores in separation distress, 25% for higher scores in dysfunctional grief, and 13% for higher scores in posttraumatic stress	na	close relationship to the deceased	na
Carson, 2021 [17]	Traumatic Stress, Coping and Post-Traumatic Growth	very high levels of post-traumatic stress, with 94.6% of the sample scoring above the threshold of 33 for a diagnosis of post-traumatic stress disorder (PTSD), levels of post-traumatic growth were very low	na	na	na
Chen, 2022 [36]	In-Person and Virtual “Goodbye”	Format of saying goodbye associated with distress and complicated grief—virtual communication and funerals as practical alternative—can arouse, rather than alleviate, more intense psychological distress	Choosing meaningful format of saying goodbye in order to make bereaved feel supported	na	na
Chen, 2021 [66]	post-traumatic stress, post-traumatic growth	Serious attention needs to be paid to the mental health issues (70%)	Grief therapies that work on the conflicts between the deceased and the bereaved and unfinished business can be applied to facilitate growth	those who lost someone younger, lost a partner or shared a close relationship	na
Downar, 2022 [41]	Bereavment after dying in acute care hospitals	Pre-COVID family members were physically present more in the last 48 h of life; the COVID + ve cohort were more present virtually, 35 family members (28.9%) had severe grief symptoms, and the prevalence was similar among the cohorts (*p* = 0.91)	na	na	na
Drucker, 2023 [21]	Depression, Complicated Grief, and Suicide Ideation	high suicidal ideation, complicated grief, and depression, suicidal ideation associated with avoidant attachment and a close relationship with the deceased	na	close relationship with the deceased	na
Gang, 2022 [43]	prolonged grief disorder (PGD)	probable PGD onset was associated with lower rates than unnatural deaths (e.g., accidents, homicide) and higher rates than natural deaths (e.g., dementia) compared to deaths from COVID-19. Less time since death, and violent causes of death were positively associated with probable PGD, while dementia was negatively associated	na	closeness of the respondent’s relationship to the deceased was positively associated with probable PGD, while extended family kinship was negatively associated	na
Harrop, 2021 [20]	Support needs and barriers to accessing support	(59%, *n* = 422) no support from bereavement services or general-Practitioner (60%, *n* = 428), when support difficulties accessing bereavement services (56%, *n* = 149)/General-Practitioner support (52%, *n* = 135), 51% reported high/severe vulnerability in grief; among these, 74% were not accessing bereavement or mental-health services, barriers included limited availability, lack of appropriate support, discomfort asking for help and not knowing how to access services, 39% (*n* = 279) experienced difficulties getting support from family/friends, including relational challenges, little face-to-face contact and disrupted collective mourning, societal strains exacerbated isolation	na	na	na
Katzman, 2022 [40]	Attachment and Emotion Regulation	differences in emotion dysregulation and attachment-related anxiety between grief reactions, the resolved grief group had the lowest levels of emotion dysregulation, importance of examining relational and emotional factors in the context of bereavement	emotion regulation capacities appear the strongest for individuals who have resolved their grief and differences in attachment-related anxiety are dependent on an individual’s grief reaction	na	na
Khalafi Kasalani, 2023 [51]	distress tolerance and relationship between existential thinking, sense of coherence, and the severity of mourning	correlation between existential thinking (*r* = 0.465), sense of coherence (*r* = 0.401), and distress tolerance (*r* = 0.521) with the severity of mourning experienced, positive and significant relationship (*p* > 0.01) between distress tolerance and sense of coherence (*r* = 0.126), as well as between distress tolerance and existential thinking (*r* = 0.059) distress tolerance did not mediate the relationship between sense of coherence and the severity of mourning	na	na	na
Lobb, 2024 [57]	Between Hospital and Home Deaths in Palliative Care	Only 37% of bereaved people received information about bereavement and support services, 38% of participants who were at least 12 months postdeath scored at a level suggestive of possible prolonged grief disorder, Levels of depression and anxiety between the two groups were not significantly different	na	na	Compared to hospital deaths, home death group had higher levels of grief severity and grief-related functional impairment
Maccallum, 2024 [58]	mental health	four classes: low symptoms (46.8%), grief (17.3%), depression/anxiety (17.7%), and grief/depression/anxiety (18.2%), the latter group reported the highest levels of health, work, and social impairment, inability to care for the deceased due to COVID-19 public health measures were correlated with grief symptoms, Preparedness for the person’s death and levels of pandemic-related loneliness and social isolation differentiated all four classes, Unemployment was associated with depression/anxiety	na	death of a child or partner was correlated with grief symptoms (with or without depression and anxiety)	na
Mayland, 2021 [22]	public health measures and individualised care	(1) public health restrictions compounding the distress of ‘not knowing’; (2) disparate views about support from doctors and nurses; (3) challenges in communication and level of preparedness for the death; (5) emotional needs and potential impact on grief	(4) delivery of compassionate care; respondents who were able to visit were associated with good perceptions of family support; timely communication and being present to ‘say goodbye’ were facilitated	na	experiencing deaths within the care home or hospital setting;
Schneider, 2023 [47]	bereaved young adults	More time spent with the deceased before the loss and greater endorsement of pandemic grief risk factors were associated with increased PGD symptoms and a greater likelihood of meeting the diagnostic criteria for PGD	na	na	na
Selman, 2022 [28]	poorer experience of end-of-life care and challenges in early bereavement	being unable to visit or say goodbye as wanted, COVID-19 was associated with worse experiences before and after death; for example, feeling unsupported by healthcare professionals, social isolation/loneliness, and limited contact with relatives/friends, expected deaths were associated with a higher likelihood of positive end-of-life care experiences	na	deceased being a partner or child increased the likelihood of positive experiences, however being a bereaved partner strongly increased odds of social isolation/loneliness	Deaths in hospital/care home increased likelihood of poorer experiences
Shahini, 2022 [55]	Comparison of Covid-19 and non-Covid-19 Causes	deceased’s burial quality caused by the Covid-19 group was significantly lower, the feeling of the Covid-19 label is significantly higher, no significant variation in grief’s broad experience between the two groups, experience of grief was significantly different in the group of relatives of the deceased infected by Covid-19 in terms of notoriety and physical reactions	na	na	na
Tang, 2021 [67]	Mental Health	severe mental health problems—feeling traumatized by the loss, and having a close and/or conflictual relationship with the deceased may elevate risk	na	Having a close relationsship	na
Wang, 2022 [23]	Gender, and Reports of depression—older adults	significantly higher probabilities of reporting depression among older adults	particular need of mental health support	older adults	na
Wang, 2022 [26]	Widowhood on Mental Health	associations between recent spousal death and poor mental health before and during the pandemic, difference-in-difference estimates indicate those whose spouses died of COVID-19 have higher risks of self-reported depression and loneliness, but not trouble sleeping, than expected based on pre-pandemic associations	na	na	na
Quantitative longitudinal surveys					
Becqué, 2023 [14]	grief experiences	Negative appreciation of the dying process (such as degrading and shocking) and “unfinished business” but not insufficient opportunity to be with the dying person were associated with higher levels of despair	na	Partners	na
Lapenskie, 2024 [46]	Long-term bereavement outcomes	prevalence of severe grief reaction remained high (28.8%) at 12–18 months post- family member death, 33.3% of family members experienced persistently high or worsening grief symptoms at the time of their 12–18-month assessment compared to baseline 6–12-month assessment, Grief severity was associated with endotracheal intubation in the deceased, but not with the cause of death (e.g., COVID vs non-COVID illness) or physical presence/absence of the family member at the bedside in the final 48 h of life	na	na	na
Quantitative and qualitative cross-sectional studies					
Majid, 2022 [63]	grief, death, mourning, and coping	Grief was found more in relatives whose loved ones died of COVID-19, Coping strategies adopted by the family members whose relative died of COVID more often adopted avoidant coping strategies as compared to non-COVID deaths, mostly, deaths occurred when the family members were not around, most of the mournings were in isolation, and there was hardly any support, most of the participants could not perform last rites which complicated the grief among them	na	grief was found more in males	na
Renckens, 2024 [24]	Experiences with and needs for aftercare ICU	na	ICU healthcare professionals may play a vital role in addressing aftercare needs by asking relatives how they are doing in the weeks following the death of their loved one and offering them a follow-up conversation with an ICU physician	na	na
Randomized controlled trial					
Stahl, 2024 [48]	Risk for Complicated Grief—Marital Partner in late life	Compared to the non-COVID-19 group, the COVID-19 bereaved group reported greater shock and disbelief, hallucinations of the deceased, and estrangement from others. COVID-19 death was also associated with higher risk for probable prolonged grief disorder (PGD) at 12 months	na	Older adults who have lost a spouse to COVID-19 present with specific symptoms of distress and may eventually require clinical care for PGD	na
Reviews					
Braz Sola, 2023 [7]	family grief	Themes: “Pandemic grief: lonely and unresolved”—interrelated and indicate that experiences of loss in this context were negatively impacted by the imperatives of physical distance, restriction of hospital visits, technology-mediated communication, and prohibition or restriction of funerals. These changes resulted in experiences marked by feelings of loneliness and helplessness	na	na	na
Firouzkouhi, 2023 [5]	Families Views	Issues raised before the death of the loved ones include no visit and absence at death time, fear of being infected with the COVID-19, death anxiety, failure to perform religious rites at death, and psychological problems. The after-death issues were related to funeral, burial, rituals, prolonged grieving, maladaptation, loneliness, and repeated mourning	na	na	na
Hasdenteufel, 2022 [35]	Psychosocial factors—palliative-stage cancer patients	Factors influencing the bereavement experience relating to: 1) the caregiver (e.g. social support, psychological burden, preparation for loss, action and discussion related to death); 2) the patient (e.g. denial or acceptance); 3) the interactions between patient and their caregivers (e.g. tensions, communication difficulties, and presence at the time of death); and 4) the end-of-life context. The caregiver’s grief experience can be described by the following terms: typical and pathological grief, anxiety, depression, guilt, psychological distress, post-traumatic stress disorder and post-traumatic growth, and life satisfaction		na	na
van Schaik, 2022 [8]	grief and bereavment	Thematic analysis showed different emotional responses, changes in grief, the effect of absence during final moments, a lack of involvement in the caring process, the impact on communities and social support systems and the alteration of funerals among bereaved relatives. During COVID-19, death is characterized by poor bereavement outcomes and health implications, but bereaved also show signs of resilience and coping		na	na
Tao, 2022 [71]	loss and grief	Loss of usual routines, lifestyles and physical health. The grief experienced was multidimensional, affecting mainly the emotional, physical, social and existential realms. Anger, guilt and fear resulted from unsatisfactory farewells, issues with funerals, social isolation, financial strain and stigmatisation	Management strategies could be categorised into 5 themes: communication, finance, counselling, education and spiritual care	na	na

## Discussion

This review identified stressors, sources of support, and risk factors for stress among bereaved individuals during the COVID-19 pandemic, focusing on participants’ experiences before and after the death of their family member. A total of 58 primary studies and five review articles were included. The studies showed that the pandemic and the associated measures placed additional stress on relatives. This finding was consistent with those of studies conducted during the COVID-19 pandemic [6–8] as well as earlier studies that examined the grieving process of relatives who lost someone in natural disasters [73, 74]. However, unique to the pandemic were the restrictions on visits during the dying phase and the restrictions on funerals, which were found to be unbearable in this review and previous studies, which for example showed a high prevalence of prolonged grief disorder as well as symptoms of depression and anxiety [9, 10]. These restrictions may have led to a unique experience, namely the lack of professional support from medical staff during the dying process and when saying goodbye, but also the lack of psychological and psychosocial services and counseling during these phases. In addition, the lack of social support from family and friends was emphasized, as social contact was prohibited or severely restricted. This may also have exacerbated feelings of isolation and loneliness, which were frequently described by the bereaved, but also by participants in studies with representative population samples during the pandemic [3, 4]. In addition to professional and social support, which was perceived as helpful, personal resources such as religion or sports and finding meaning were described as helpful. This was also found in other studies on the COVID-19 pandemic and in studies on grief after natural disasters [7, 11, 75, 76].

Future research on dying and mourning in general, but also in times of crisis, should always consider and analyze stress factors and supportive factors together. In addition, studies on the long-term consequences for people who have lost a loved one during the COVID-19 pandemic would be extremely desirable and important. Further studies using mixed methods could also enrich the research, as the results of both approaches are highly relevant and it would be desirable to combine them in primary studies.In terms of practical implications, it would be important to develop concrete measures based on the findings of pandemic research to support relatives immediately after death. Networks of hospitals, care facilities, counseling centers, and psychotherapeutic institutions could be established or expanded for this purpose. It is also important to have clearly defined referral processes that include plans for times of crisis. One focus here should be on ensuring that staff are flexible, so that existing or prescribed measures are not enforced as a matter of principle, but can be reviewed on a case-by-case basis and applied accordingly. This could include working together with relatives to find individually tailored solutions [77]. In addition, alternative means of communication between medical staff and relatives via digital devices should be expanded and offered not only during pandemics. These should be considered as an option, but with the awareness that they often do not convey the same sense of connection. The results of the study also show that social support for those affected needs to be strengthened. A first step toward promoting this could be the involvement and participation of (former) relatives in the development of appropriate measures. In this way, the specific concerns and experiences of those affected can be taken into account and perspectives can be incorporated into the development of measures and networks that the staff (medical, nursing, psychosocial, psychological) do not have. In addition, professional support should focus on discussing individual options for social support and offering networking opportunities, as well as addressing feelings of loneliness among the bereaved and, if necessary, referring them to professional psychological help..

## Limitations

It is possible that the search strategy described above may have overlooked some literature, which could have resulted in important topics not being identified or gaps arising. However, the search strategy was thoroughly discussed with all authors and trained librarians were consulted to develop a search strategy that minimizes the possibility of overlooking literature. This overview also does not include an assessment of the quality of the included studies, as they are very heterogeneous in terms of methodology. In order to do justice to all methodological approaches and their qualities, the quality assessment would have to be so detailed and comprehensive that it would go beyond the scope of a scoping review. The broad inclusion criteria made it difficult to compare the studies. For example, studies that included not only relatives but also friends of the deceased were included. This could limit the validity of the results with regard to the specific burdens and support options for bereaved relatives. In addition, only studies in English and German were considered. Studies in other languages might have provided more comprehensive results. It is important to note that some of the issues described in this review were certainly problematic for bereaved relatives even before the pandemic, but no comparisons were made in this area and the studies focused in particular on the restrictions imposed by the pandemic and their consequences.

## Conclusion

In summary, it can be said that the COVID-19 pandemic, and in particular the restrictions on end-of-life care and mourning rituals following the death of a loved one, placed an additional burden on the bereaved and exacerbated the mental health problems of those left behind. Feelings of isolation and loneliness were widespread, and bereaved individuals reported a lack of professional and social support. Meaning-making, religion, spirituality, and flexibility in professional and social support were cited as resources. Consequently, both professional support and structures to promote social support during pandemics must be prioritized in preparations for future crises. To this end, the government must provide financial resources in particular so that networks of hospitals, funeral homes, psychosocial and psychological institutions, counseling centers, and those affected can jointly develop and implement concrete services and processes for providing these services.

## Data Availability

No datasets were generated or analysed during the current study.
